# Microleakage of porcelain laminate veneers cemented with different bonding techniques

**DOI:** 10.4317/jced.53954

**Published:** 2018-02-01

**Authors:** Satheesh B. Haralur

**Affiliations:** 1 Associate Professor, Department of Prosthodontics, College Of Dentistry, King Khalid University, Kingdom of Saudi Arabia

## Abstract

**Background:**

Porcelain laminate veneers (PLV) are continued to be popular in modern dental practice due to its high aesthetic outcome and conservative tooth preparation. The composite resins are commonly used as luting agents for cementation of PLV. Polymerisation shrinkage induced marginal gap and interfacial leakage is the persistent limitation with the resin luting cements. The objective of the study was to assess the effect of various dentin bonding techniques on the microleakage after accelerated ageing in porcelain laminate veneres.

**Material and Methods:**

Forty intact, premolar teeth were prepared to receive the PLV. The lithium disilicate PLV were fabricated from IPS e.max System. The intaglio surfaces were prepared with hydrofluoric acid and silane. Teeth samples were randomly divided among four groups of ten each according to the tooth surface preconditioning; it included etch-wash light cure, etch-wash dual cure, self-etch and self-adhesive techniques. The Teeth samples were subjected to the accelerated ageing with thermocycling and exposure to xenon light. The microleakage was accessed with die penetration test using 0.5% basic fuchsin. The data was statistically analysed by Kruskal–Wallis and Mann–Whitney U tests.

**Results:**

The etch-wash dual cure resin cements recorded the lowest interfacial microleakage score of 0.90 and 0.60 at cervical tooth-composite resin and incisal enamel-composite regions respectively. The highest corresponding values of 3.00 and 1.60 was recorded with self-Adhesive resin luting cements. The microleakage at cervical tooth- composite interface was significantly higher in comparison to incisal enamel-composite interface across all the tested groups. The microleakage values at porcelain-composite interface was considerably less to the tooth- composite interface.

**Conclusions:**

Etch-wash composite resin luting cements for PLV provided the best bonding interface, with the least interfacial microleakage.

** Key words:**Porcelain laminate veneer, Microleakage, Tooth conditioning, Bonding techniques.

## Introduction

The dramatic increase in use of ceramic veneer is experienced in contemporary dentistry due to the aesthetic conscious society. Porcelain laminate veneers (PLV) are conservative aesthetic treatment of choice to restore the malaligned, worn, fractured and discoloured teeth. The ceramic veneer has multiple advantages over the direct composite restoration like better aesthetics, colour stability, wear resistance and thermal expansion similar to natural teeth. The PLV tooth preparation is conservative in comparison to full veneer ceramic crowns. The challenge is to fabricate the ceramic laminate veneer closely adapted to the prepared tooth surface. The previous studies evaluating the marginal fidelity in porcelain veneer reported the marginal gap between the ceramic veneer and prepared tooth in the range of 60- 292µm (1). The marginal integrity with minimum space reduces the resin luting cements volume.

The advancement in bonding technique like ceramic etching (2), silane application (3) and enhanced physical properties of resin luting agents have improved the bonding strength between ceramic veneer and prepared teeth. The composite resin luting cements are reported to undergo the polymerisation shrinkage in the range of 2.6% -5.7 % (4). The polymerisation shrinkage induced stress is expected to create a marginal gap between the ceramic veneer and tooth structure. The studies have also recorded the dissolution of the exposed resin cement in the margin, possibly lead to the micro-gaps. The difference in the Coefficient of thermal expansion between, natural teeth, ceramic and composite resin may also encourage the development of microscopic gap (5). The ceramic veneer bonded to tooth with composite resin cements produces two bonded interfaces. One between ceramic – composite resin cement and other between the tooth- composite resin interface. The polymerization and thermal expansion process induced stress lead to the counteractive competition between two bonded interfaces, and resulting in debonding at the interface with lowest the adhesive strength. Microleakage is considered as important criteria to assess the long-term success of restorative material, and it is defined as the chemically undetectable passage of bacteria, fluids, molecules or ions between the cavity walls and the restorative materials (6). The bonding between the dentin and the composite is less predictable in comparison to enamel surface (7).

The partial dentin demineralisation, higher percentage of organic tissue and the presence of dentinal fluids explain the lesser predictability of bonding to the dentin.

Various bonding techniques are developed over the years to advance the bonding strength and to reduce the microleakage. The light activated composite resin luting cement is preferred due to its longer working time and better colour stability. The dual cure cements are preferred by few clinicians owing to higher physical property attributed to a higher degree of polymerisation (8). It is also favoured in the case of PLV thickness more than 0.7mm (9). The dentin conditioning is made simpler by introduction of self-etch and self-adhesive resin cements. The previous studies are contradictory in their finding about the effect of dentin bonding agents on the microleakage. Zaimoglu A *et al.* (10) described the substantial reduction in microleakage while Sim *et al.* (7) reported the non-significant reduction in microleakage over the use of third-generation bonding agent. The enamel-dentin conditioning mostly include etch- wash, self-etch and self-adhesive techniques. There is considerable difference in the clinical procedure, component and bonding chemistry between these bonding techniques. The researchers report the disparity in degradation process between these bonding agents on ageing. The biodegradation of resin-dentin bonds in etch-wash system includes the collagen hydrolysis while the hydrolytic degradation at the composite/ adhesive junction is predominantly observed in self-etching adhesives (11). Though the microleakage in different resin cements is extensively studied, the comparative evaluation of microleakage between different bonding techniques still needs further investigation. Hence this *in-vitro* study was planned to assess the effect of various dentin bonding techniques on the microleakage after accelerated ageing in ceramic laminate veneers.

## Material and Methods

Total of forty intact sound maxillary premolar teeth were included in this *in-vitro* study. The teeth were extracted for orthodontic or periodontal reasons and stored in physiologic saline solution. The exclusion criteria included the caries, restorations, cervical abrasions, micro- cracks and hypo calcified lesions. The extracted teeth were cleaned with hand scaling to remove the calculus and examined under X 2.5 magnification to assess the micro cracks. The teeth samples were mounted on the auto polymerising acyclic block to improve the control during the teeth preparation.

The horizontal grooves of 0.5mm were placed with depth preparation diamond bur. All teeth samples were anatomically reduced with medium grit round ended diamond bur (diatech, coltane. AG, Switzerland). The mesial and distal proximal extensions were placed up to the contact area. The cuspal reduction of 1.5 mm was included to create the incisal overlap .

-Fabrication of the porcelain veneers

Lithium disilicate glass ceramic (IPS e.max, Ivoclar, Schaan/Liechtenstein) PLV were fabricated by burnout/ heat pressing of wax pattern at 9200C. The PLV were glazed by baking them at 7650C. The Intaglio surfaces of the PLV were etched with 5 % hydrofluoric acid (IPS ceramic etching gel) for 20 seconds. The samples were cleaned in ultrasonic bath for 10 minutes and dried with oil-free air. The fitting surfaces were silanated (Monobond Plus) and allowed to react for one minutes.

The teeth samples were randomly divided into four groups of 10 each according to the conditioning of corresponding tooth surface.

Group I: etch-wash light cure ( Variolink Veneer, Ivoclar Vivadent AG, Bendererstrasse, Liechtenstein): The tooth surface was etched with 37% phosphoric acid for 20 seconds. The surface was rinsed with water, and blot dried. The self-priming bonding agent applies for 20 seconds and light cured for 20 seconds.

Group II: Self-Etch: (Panavia F 20, Kuraray Medical Inc, Okayama, Japan). Equal amount of primer A and B was mixed, applied over the tooth surface and allowed to cure for 20seconds. The paste A and B was mixed in identical proportion and applied to the fitting surface of PLV. After seating, it was light cured for 20 seconds and oxyguard was applied at the exposed margins.

Group III: Etch-Wash- Dual cure (Rely X ARC, 3M ESPE, St. Paul, USA ) : Tooth conditioning was similar to the group I, except the adhesive was dual-cure in nature.

Group IV: Self- Adhesive (Rely X unicement, 3M ESPE, St. Paul, USA). No pre-conditioning of tooth surface; the cement capsules were activated and mixed for 15 seconds in amalgamator. The cement was applied PLV, the allowed to self-cure for 2-3 minutes followed by the light-cure for 20seconds.

-Assessment of microleakage

**Figure 1:**
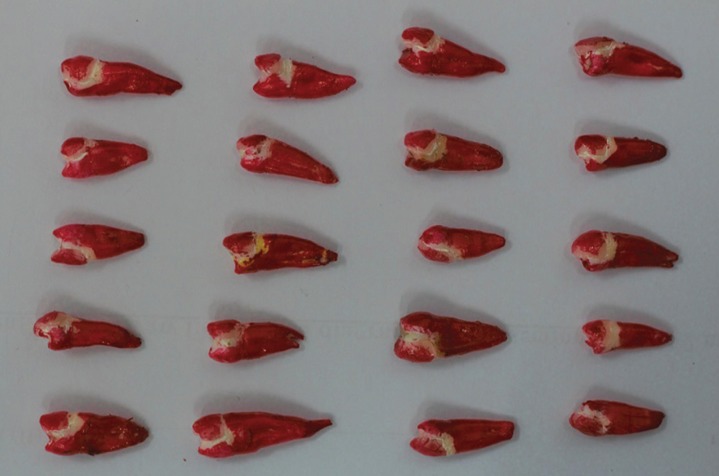
Tooth samples coated with two coats of nail varnish.

The teeth samples were diligently demounted from acrylic blocks, the root apices were sealed with glass ionomer cement. The teeth samples were stored in distilled water for 30 days at a controlled temperature of 370C. The teeth samples were subjected for thermocycling between 50C and 550C for 5000 cycles with a dwelling time of 30 seconds and 20 seconds travelling time. The samples were exposed to xenon lamp of 70000K for 100 hours with controlled temperature of 370C with 100 % humidity. All tooth surfaces except for 1 mm around the veneer margin were coated with two coats of nail varnish (Fig. 1). The teeth samples were immersed in 0.5% basic fuchsin dye for 24 hours. The teeth samples washed thoroughly under running water and lightly pumiced to remove the dye on surface area. Each teeth sample was sectioned at the centre longitudinally in facio-lingual direction with the help of 0.5 mm low speed diamond disk.

**Figure 2:**
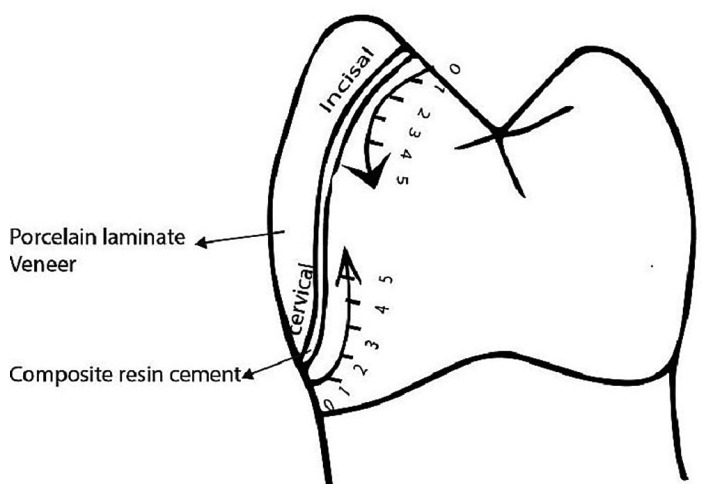
Criteria for evaluation of microleakage.

**Figure 3:**
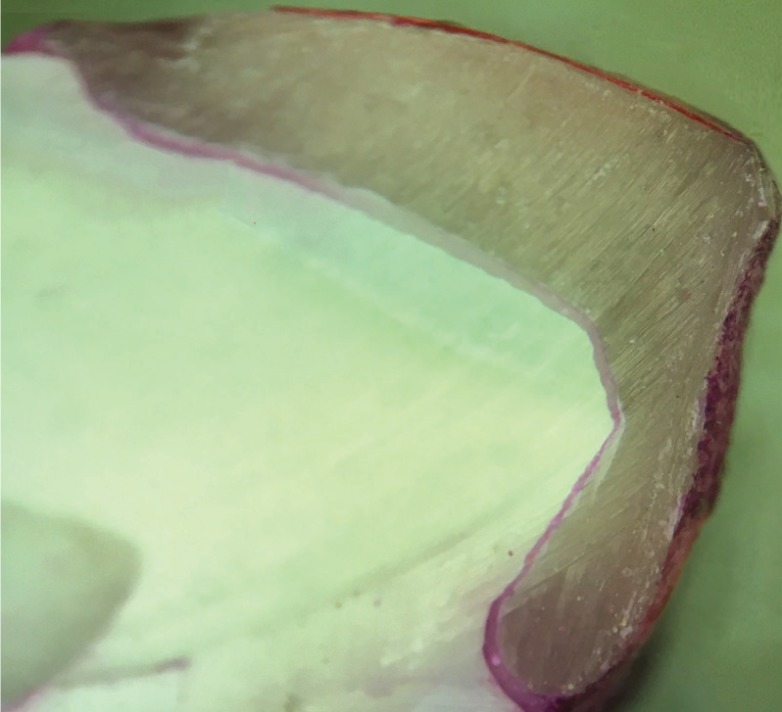
Image displaying the die inflitration.

Both hemi sections were evaluated with the help of a stereomicroscope at ×30 magnification. Following criteria were observed for scoring microleakage in all four interfaces (10) (Fig. 2): no penetration of dye (0); penetration of dye up to one-fifth (1); two-fifths (2); three-fifths (3); and four-fifths (4) of the cervical or incisal margin. Penetration of dye along the entire cervical or incisal margin was denoted as 5 (Fig. 3). The obtained data was analysed with SPSS software (IBM Corporation, Armonk, New York, USA). The Kruskal–Wallis and Mann–Whitney U‑tests were conducted for the evaluation of microleakage between groups with the level of *P* = 0.05 significance.

## Results

The mean microleakage at different interfaces are detailed in the  Table 1. The mean microleakage for cervical tooth composite resin was least with group III (etch-wash dual cure) with a score of 0.90. It was followed by Group I etch-wash light cure resin at 1.70 and highest marginal leakage was observed with Group IV (self-adhesive) with the score of 3.00. The incisal enamel- composite interface microleakage showed less microleakage in comparison to the cervical area across all the groups. However microleakage scores among the groups showed the similar tendency like cervical-composite interface. Least interface microleakage at incisal enamel-composite resin of 0.60 was recorded with etch-wash dual- cure (Group III) and higher value of 1.60 was recorded with self-Adhesive (Group IV) cements. The microleakage at porcelain- composite resin interface at the both cervical and incisal regions was substantially less in comparison to tooth- resin interface. The least microleakage value at porcelain- composite interface was recorded by Group IV in both cervical and incisal areas.

**Table 1:**
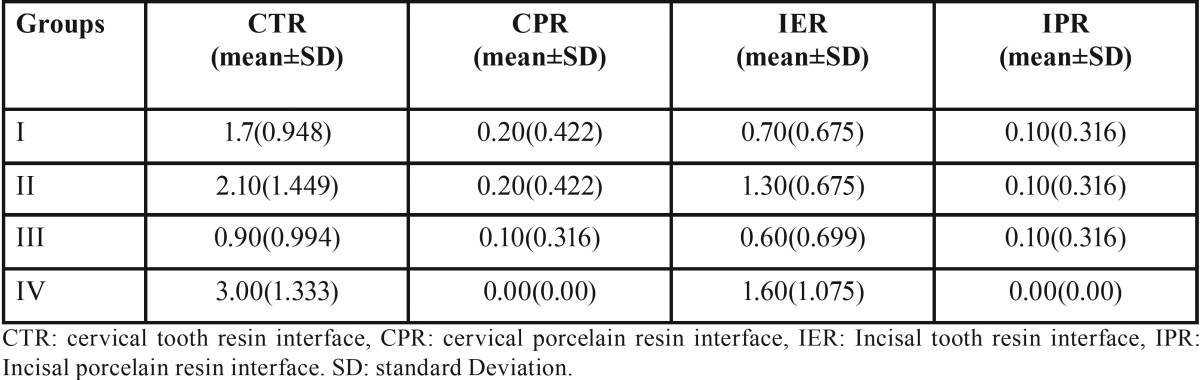
Mean interfacial leakage scores.

The Kruskal–Wallis test ( Table 2) was performed to examine the dissimilarity in mean ranks between the investigated groups. The analysis indicated the presence of a significant difference in mean ranks between the groups at cervical tooth-composite interface with the *p* value of 0.009. The Mean ranks were also significantly different at incisal enamel – composite resin interface with a *p* value of 0.037. The mean rank difference between the investigated groups was non-significant at porcelain- composite resin interface in the both cervical and incisal areas (*P* ≥ 0.05). The lowest mean value rank was recorded for group III with rank of 11.85 and 14.80 at cervical tooth-composite resin and incisal tooth-composite resin interface respectively. It was followed by Group I, Group II and Group IV at cervical tooth-composite resin interface with mean rank of 19.05, 22.15 and 28.95 correspondingly. The similar mean rank pattern was also observed for incisal enamel- composite resin interface.

**Table 2:**
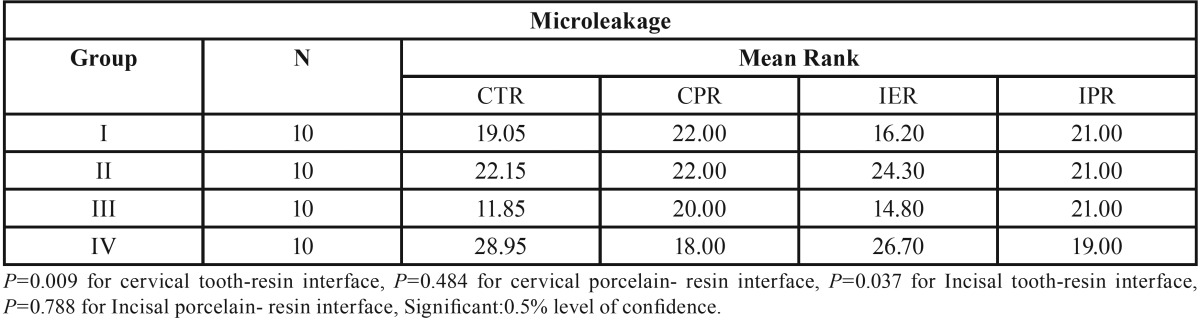
Kruskal-Wallis analysis for mean microleakage of various groups at different interfaces.

The pair-wise Mann-Whitney test ( Table 3) was conducted to identify the dissimilarity between individual groups. The results indicated the presence of statistically significant difference between Group I and Group IV at tooth- composite resin interface in both cervical and incisal location. The *p* values between these groups at cervical and incisal locations were 0.030 and 0.048 respectively. The result confirms the statistically significant difference between Group III and Group IV with a *p* value of 0.003 and 0.033 for tooth-resin interface at cervical and incisal area respectively.

**Table 3:**
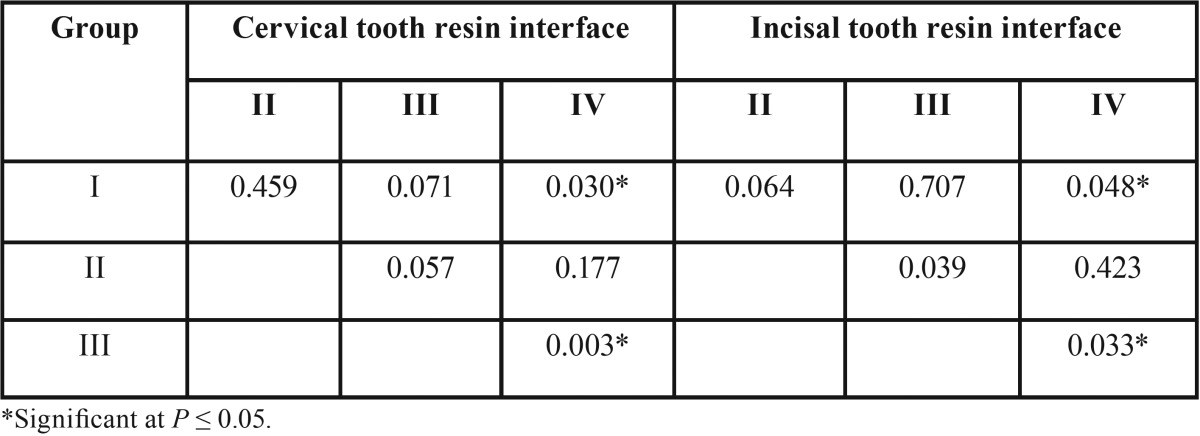
Pair‑wise comparisons using Mann–Whitney tests for each group at cervical tooth resin interface and Incisal tooth resin interface.

## Discussion

The PLV combines the both advantages of high aesthetics and conservative treatment. The researchers report the encouraging clinical survival rate of 94.4 -92% at five years and 93.5 -64% at 10 years (12) for PLV restorations. The primary reasons for clinical failures of PLV include the fractures, debonding, large marginal defects and discolorations. The interfacial leakage is ascribed to the partial debonding, discoloration, and fracture (13). The polymerisation shrinkage of resin cements and the differences in coefficients of thermal contraction of bonded surfaces are credited for the accelerated marginal gap formation (14). The researchers advocated various methods to evaluate the microleakage, like dye penetration, bacterial leakage, radio isotopes infiltration and dye extraction. The dye penetration method provides the information of internal seal by luting cement, and it also allows the direct observation of dye penetration under magnification. Hence, this method was utilised during the study to access the microleakage. Furthermore, the molecular weight of the basic fuchsin is less than the average diameter of oral bacteria cell, hence it is helpful in detecting the small marginal discrepancy. Majority of earlier researches on the microleakage over the PLV included only the thermocycling during accelerated ageing. The laminate veneers in aesthetic zones are constantly exposed to the visible light. Therefore, the samples were exposure to xenon light to simulate the complete accelerated ageing procedure according to ISO standards (15).

The results of the study indicated the microleakage at the tooth- composite resin interface at cervical area was significantly higher than the enamel-composite resin interface across all the Groups. The findings from the study are in corroboration with results reported by Tjan *et al.* (16) and Beznos *et al.* (17). The researchers suggested the enamel rod orientation at the cervical area may lead to increased microleakage at cervical interfaces. The results from the study also showed the microleakage was predominant at tooth- composite interface in comparison to porcelain- composite interface. The etched enamel or dentin surface has less micro-topical irregularity in contrast to the etched porcelain surface. Hence, porcelain with it larger surface irregularity provides the stronger micro-mechanical strength. Stacey *et al.* (18) reported the excellent adhesive strength between etched silanised porcelain/ composite resin (33Mpa), and it was significantly higher than the composite /etched enamel bond strength (31Mpa). The contraction stress is induced during the polymerisation shrinkage, resulting in the micro-gap formation at weaker bond interface (19).

The tooth- composite resin interface microleakage at the cervical and incisal area was recorded highest for the self- adhesive cement with a score of 3.00 and 1.60 respectively. The microleakage was recorded least by the etch-wash resin luting cements. The dual cure etch-wash resin cement showed the microleakage score of 0.90 at cervical location and 0.60 at incisal enamel- composite interface area. The results are in concurrence with observation from earlier studies. Bott *et al.* (20) stated the good marginal adaptation was observed in dual cure resin cement and chemical cure resin cements. Frankenberger *et al.* (21) reported the poor margin integration with self- adhesive cement after thermo-cycling as a luting agent for ceramic inlays. Ibarra *et al.* (22) during the microleakage evaluation for self –adhesive cement showed the higher microleakage. Hence, he suggested the separate etching procedure prior the PLV cementation with self- adhesive cement. Few other researchers like, Rosentritt *et al.* (23) reported the similar marginal adaption between self-adhesive, self-etch and etch wash resin cements. Shafiei *et al.* (24) concluded the best enamel sealing of the cemented veneers was obtained with the etch-rinse resin luting cements. The self-adhesive cements are comprised of filled polymers designed to adhere to tooth structure without any pre-conditioning of enamel or dentin. The Self-adhesive resin cement composed of mono-multi methacrylate monomers in variety of resin based dental materials like Bis-GMA, UDMA, HEMA. The acid functionalised monomers are used to attain demineralisation and bonding to the tooth structure (25). The concentration of the acidic monomers is balanced to avoid hydrophilicity, while keeping it high enough to obtain adequate self-etching. The hydrophilic nature of acid monomers causes the swelling and consequently, compromises the mechanical strength and dimensional stability (26). The smear layer is enveloped into the bond structure in both self-etch and self-adhesive resin cements. The hybrid layer is composed of resin, collagen fibrils and minerals. Incomplete infiltration of primers within the hybrid layer allows the nano-leakage. It induces the formation of water tree’s, bubbles and phase separation of bond faces (27). The hydrophilic nature of bonding resins leads to water absorption and replacement of hydrophilic monomers even after curing. This process results in the hydrolytic degradation of bonding surface over long term (28). Total etch-wash luting cements employs the acidic solutions like 35 % phosphoric acid for demineralisation of smear layer and it exposes the collagen matrix. The collagen hydrolysis is mainly due to inadequate infiltration of exposed collagens, incomplete encapsulation of demineralised dentin from the adhesive resin (11). The matrix metalloproteinases(MMP) are also attributed for the degradation of exposed collagen fibres (29). The polymerisation of the light cure resin depends upon multiple factors like thickness, shade of PLV and light source. The contraction of resin towards the light source may disrupt the weaker tooth- resin bond interface. Hence it could result in higher microleakage values in etch-wash light cure resin luting cements. The limitation of the study includes the role of functional load was not considered during the microleakage evaluation. The thickness of the luting cement plays an important role in determining the stress due to polymerisation shrinkage. Hence further researches are required to evaluate the effect of luting cement thickness over the microleakage in PLV fabricated from different bonding techniques.
